# CXCR7 Inhibits Fibrosis via Wnt/*β*-Catenin Pathways during the Process of Angiogenesis in Human Umbilical Vein Endothelial Cells

**DOI:** 10.1155/2020/1216926

**Published:** 2020-06-05

**Authors:** MinQian Shen, YiFan Feng, Jing Wang, YuanZhi Yuan, Fei Yuan

**Affiliations:** Department of Ophthalmology, Zhongshan Hospital, Fudan University, Shanghai 200032, China

## Abstract

Although SDF-1/CXCR7 plays an important role in angiogenesis, the function and the pathway of the SDF-1/CXCR7 axis might depend on the cell type or tissue origin and not fully understood. In this study, we investigated the effect of CXCR7 in SDF-1-induced proliferation, migration, apoptosis, tube formation, and endothelial-to-mesenchymal transition (EndMT) of human umbilical vein endothelial cells (HUVECs), and the potential pathway of SDF-1/CXCR7. We confirmed that the silencing of CXCR7 inhibited the proliferation of HUVECs and contributed the apoptosis, while overexpressed CXCR7 increased SDF-1-induced HUVECs migration and tube formation. However, upregulated CXCR7 inhibited the expression of *α*-SMA, suggesting that CXCR7 might attenuate EndMT. In addition, overexpressed CXCR7 activated AKT and ERK signaling pathways but suppressed Wnt/*β*-catenin pathways in HUVECs. The inhibition of Wnt/*β*-catenin pathways decreased the expression of *α*-SMA. Altogether, these results suggest that CXCR7 might inhibit fibrosis via Wnt/*β*-catenin pathways during the process of angiogenesis.

## 1. Introduction

Angiogenesis is a key component of many pathological conditions, including corneal neovascularization (CorNV). Corneal neovascularization, resulting from a variety of etiologies, is a sight-threatening pathological change and part of a wound healing response culminating in fibrosis and scar formation [1, 2]. Although the mechanism of CorNV has been unclear, the mounting evidence shows that lymphangiogenesis and angiogenesis are closely related to CorNV [3]. Chemokines, including stromal-derived factor 1 (SDF-1), are small molecules for leukocyte migration and recruitment [4] and have been reported involved with lymphangiogenesis and angiogenesis [5].

SDF-1, also known as CXCL12, has been confirmed to be associated with pathological angiogenesis including CorNV [6, 7]. For many years, CXCR4 has been considered the exclusive receptor for SDF-1 until CXCR7 was discovered [8, 9]. CXCR7 is involved with the proliferation, invasion, migration, and adhesion of several cancer cells [10–18] and plays a role in the pathophysiological processes of diabetic limb ischemia [19], cerebral ischemia [20], coronary artery disease [21], and vascular eye diseases [22–26]. The previous literature shows SDF-1/CXCR7 axis can not only activate MAPK/ERK [21, 27–29] and PI3K/AKT pathway [13, 15, 19, 30], but also STAT3 [31] and Notch pathway [32]. Thus, the function and the pathway of the SDF-1/CXCR7 axis might depend on the cell type or tissue origin [26]. In our pilot study [23], we found that TC14012, a CXCR7 agonist, initially enhanced alkali burn-induced CorNV, but this effect did not maintain over time. The results indicated that CXCR7 might play a role in the pathogenesis of CorNV in addition to CXCR4, but the mechanism was not fully understood.

The cornea is transparent and devoid of blood and lymphatic vessels. In certain pathological conditions, the corneal host cells, including corneal epithelial, stromal, and endothelial cells can play intricate roles in angiogenesis via releasing series factors, which shift the balance toward CorNV [33]. Then, the process of neovascularization includes the activation, proliferation, and migration of endothelial cells, and the formation of vascular tubes and networks [34]. Therefore, endothelial cells are the effector cells. Human umbilical vein endothelial cells (HUVECs) exhibit key endothelial cell phenotypes [35] and are widely used in the in vitro research of CorNV. In the current study, we determined the role of CXCR7 in angiogenesis and fibrosis as well as the potential pathway of SDF-1/CXCR7 axis in HUVECs.

## 2. Materials and Methods

### 2.1. Cell Culture

HUVECs (Jennio Biotech, Guangzhou, China) were maintained in DMEM/F12 medium (Biological Industries, Beit Haemek, Israel) containing 1% endothelial cell growth supplement (ECGS, Sciencell, CA, USA), 20% fetal bovine serum, 5 U/ml of penicillin, and 5 mg/ml of streptomycin and were used at passage 8-10.

### 2.2. CXCR7 Knockdown and Overexpression

To knockdown CXCR7 expression, specific siRNAs against human CXCR7, alone with the negative control, were transfected into HUVECs using Lipofectamine 3000 (Thermo Fisher, Waltham, MA). We selected 3 target sequences for CXCR7 and the negative control as follows: CXCR7-siRNA 1, forward (GCUAUGACACGCACUGCUATT), and reverse (UAGCAGUGCGUGUCAUAGCTT); CXCR7-siRNA 2, forward (GCAGCCGGAAGAUCAUCUUTT), and reverse (AAGAUGAUCUUCCGGCUGCTT); CXCR7-siRNA 3, forward (GCUUCAUCAAUCGCAACUATT), and reverse (UAGUUGCGAUUGAUGAAGCTT); negative control, forward (UUCUCCGAACGUGUCACGUTT), and reverse (ACGUGACACGUUCGGAGAATT). After transfection for 48 h, the expression of CXCR7 was determined by quantitative real-time PCR and western blot.

To upregulate the CXCR7 expression in HUVECs, the plasmid encoding overexpressed CXCR7 was constructed by cloning the CXCR7 gene into XhoI and EcoRI sites on pEX-1 vector (Shanghai GenePharma, Shanghai, China). The CXCR7 cDNA was amplified using the following primers: forward (CGCAAATGGGCGGTAGGCGTG) and reverse (TAGAAGGCACAGTCGAGG). HUVECs were transfected with recombinant CXCR7 or control vector using Lipofectamine 3000 overnight. After transfection for 48 h, the efficiency was determined by quantitative real-time PCR and western blot.

### 2.3. Quantitative Real-Time PCR (qRT-PCR)

Total RNA was extracted using Trizol reagent (Invitrogen, Carlsbad, CA, USA) and reversed transcribed with PrimeScript® RT Master Mix (TaKaRa, Otsu, Japan). qRT- PCR was performed using the ABI Prism 7700 sequence detection system (Applied Biosystems, Foster City, CA, USA) and using the SYBR Premix Ex Taq™ II Kit (Takara) according to the manufacturer's instructions. The primes were as follows: CXCR7, forward (ACGTGGTGGTCTTCCTTGTC), and reverse (AAGGCCTTCATCAGCTCGTA); GAPDH, forward (CTCAGACACCATGGGGAAGGT), and reverse (TGATCTTGAGGCTGTTGTCATA). Relative mRNA expression levels were calculated by the 2^-*∆∆*Ct^ method. GAPDH was used as a reference gene.

### 2.4. Western Blot

The cells were lysed in RIPA Lysis Buffer (Beyotime, China) supplemented with protease inhibitors. The total protein concentration was measured using a BCA protein assay kit (Pierce, MA, USA) according to the manufacturer's instruction. A total of 20 *μ*g protein was separated on 10% SDS-PAGE and transferred onto PVDF membranes (Beyotime). Blocking was performed in 5% nonfat dried milk in Tris-buffered saline containing 0.1% Tween 20 at room temperature for 2 hours. The membranes were incubated overnight at 4°C with primary antibodies, including the following: polyclonal anti-CXCR7 (1 : 400; Abcam, Cambridge, MA, USA), polyclonal anti-pan-AKT (1 : 1000; Abcam), polyclonal anti-phospho-AKT (1 : 500; Abcam), monoclonal anti-ERK1/2 (1 : 5000; Abcam), monoclonal anti-phospho-ERK1/2 (1 : 1000; Abcam), monoclonal anti-*β*-catenin (1 : 400; Abcam), monoclonal anti-c-Myc (1 : 500; Abcam), polyclonal anti-cyclinD1 (1 : 1000; Abcam), polyclonal anti-survivin (1 : 5000; Abcam), monoclonal anti-*α*-SMA (1 : 1000; Abcam), and monoclonal anti-GAPDH (1 : 10000; Abcam). The membranes were then washed with TBST three times, incubated with HRP-conjugated secondary antibody (Jackson ImmunoResearch, West Grove, PA, USA) for 2 hours at room temperature, then detected with ECL detection reagents (Thermo Fisher Scientific, Waltham, MA, USA). GAPDH was used as a loading control.

### 2.5. Cell Proliferation Assay

After CXCR7 siRNA or overexpressed CXCR7 transfection for 48 h, the HUVECs were seeded into 96-well plate (3000 cells/well). Cell proliferation was detected after SDF-1 (100 ng/ml) treatment for 24 h with CCK-8 kit (Dojindo, Kumamoto, Japan) according to the manufacturer's instruction. The optical density was measured with a microplate reader at 450 nm.

### 2.6. Flow Cytometry Analysis of Cell Apoptosis

Apoptosis was detected by annexin V-FITC kit (Beyotime). In brief, HUVECs were transfected with CXCR7 siRNA or overexpressed CXCR7 for 48 h, then cultured with SDF-1 (100 ng/ml) for 24 h. After experimental treatment, HUVECs were collected and washed 2 times, then incubated with annexin V-FITC and propidium iodide (PI) for 15 min at room temperature in the dark. Cells were then immediately analyzed by flow cytometry.

### 2.7. Transwell Migration Assay

Cell migration assay was performed in a 6-well plate with 8.0 *μ*m pore-size transwell inserts (Corning, Corning, NY, USA). The upper surface chamber was pretreated with Matrigel (Corning) and serum-free DMEM/F12 (1 : 8) medium. After CXCR7 siRNA or overexpressed CXCR7 transfection for 48 h, a total of 1 × 10^5^ HUVECs were seeded to the upper chamber with serum-free DMEM/F12 media. The lower chamber was filled with 500 *μ*l DMEM/F12 medium containing SDF-1 (100 ng/ml) and 10% FBS. After incubation at 37°C for 24 h, the cells that migrated to the lower membrane surface were fixed with 4% paraformaldehyde and stained by 0.1% crystal violet. The number of cells in randomized 5 fields was counted under a microscope.

### 2.8. Scratch Wound Assay

A total of 1.5 × 10^4^ upregulated or downregulated CXCR7 HUVECs were seeded onto a 6-well plate for 24 h, and then exposed to SDF-1(100 ng/ml). A wound was created after manually scraping the cell monolayer with a p200 pipet tip. The initial wound quantification was performed on images collected at 0 hour after wounding, and further images were collected randomly in wound areas at 18 h after wounding. The wound width was measured, and the migration was represented as percentage migration considering migration in untreated control as 100%.

### 2.9. Tube Formation Assay

Aliquots (50 *μ*l) of Matrigel (Corning) were added to a 96-well plate and were incubated at 37°C for 1 h. HUVECs were pretreated as below: (1) control; (2) SDF-1 (100 ng/ml); (3) SDF-1 and siRNA negative control transfection; (4) SDF-1 and CXCR7 siRNA transfection; (5) SDF-1 and over-expression control; (6) SDF-1 and CXCR7 overexpression transfection. The cells were resuspended in supernatants collected from each pretreatment and then seeded onto the gel (2 × 10^4^ cells/well). Five random fields from each well were chosen and photographed after 4 h. Networks of tube-like structures were measured using Image-Pro Plus software, version 6.0 (Media Cybernetic, Silver Spring, MA, USA).

### 2.10. Immunofluorescence Staining

HUVECs were pretreated as before. The cells were fixed with 4% paraformaldehyde in 1 × PBS for 15 min at room temperature, blocked with PBS/0.3% Triton™ X-100 for 1 h. Then, cells were incubated with anti- *α*-SMA (1 : 500; Abcam) overnight at 4°C. After 3 washes in PBS, cells were incubated for 1 h with Alexa Fluor® 488-conjugated secondary antibody(1 : 1000; Abcam). Cells were rinsed in PBS and incubated with DAPI for 5 min. Images were captured with an inverted microscope (Leica, Wetzlar, Germany).

### 2.11. Statistical Analysis

The data were presented as the mean values of 3 or 4 independent experiments. Student's *t* test and one-way ANOVA analysis were performed using SPSS 20.0 (SPSS, Chicago, Ill, USA) for all statistical data. All analyses were carried out using GraphPad Prism (GraphPad Software, La Jolla, CA). Values were expressed as the mean ± SD, and statistical significance was set at *p* < 0.05.

## 3. Results

### 3.1. The Downregulation and Upregulation of CXCR7 in HUVECs

**Figure 1:**
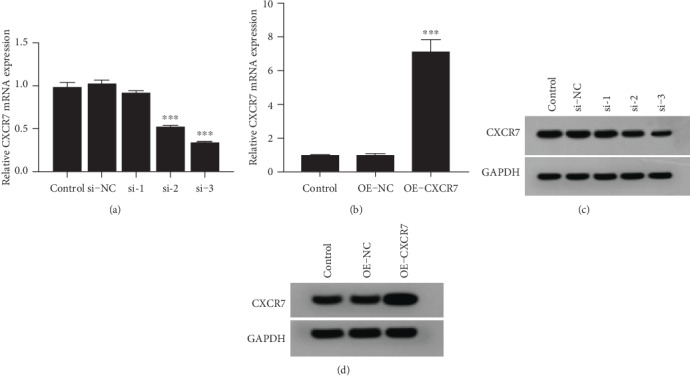
The downregulation and upregulation of CXCR7 in HUVECs. (a, c) The mRNA expression of CXCR7 was detected by qRT-PCR in HUVECs transfected with CXCR7-siRNA and overexpressed CXCR7 plasmid vector. (b, d) Western blotting analyzed levels of CXCR7 in HUVECs transfected with CXCR7-siRNA and overexpressed CXCR7 plasmid vector. si-NC: siRNA negative control group. OE-NC: overexpression negative control group. ^∗∗∗^*p* < 0.001 versus untreated control group.

After selection with puromycin, the expression of CXCR7 in HUVECs was detected by qRT-PCR and western blotting. The level of CXCR7 mRNA and protein in HUVECs transfected with CXCR7-siRNA 3 was decreased (*p* < 0.001) (Figures 1(a) and 1(b)). On the contrary, the level of CXCR7 mRNA was significantly increased with overexpressed CXCR7 plasmid vector transfected (*p* < 0.001) (Figures 1(c) and 1(d)). These results indicated that CXCR7 knockdown and overexpressed HUVECs could be available to further researches.

### 3.2. The Effects of CXCR7 on the Proliferation and Apoptosis of HUVECs

**Figure 2:**
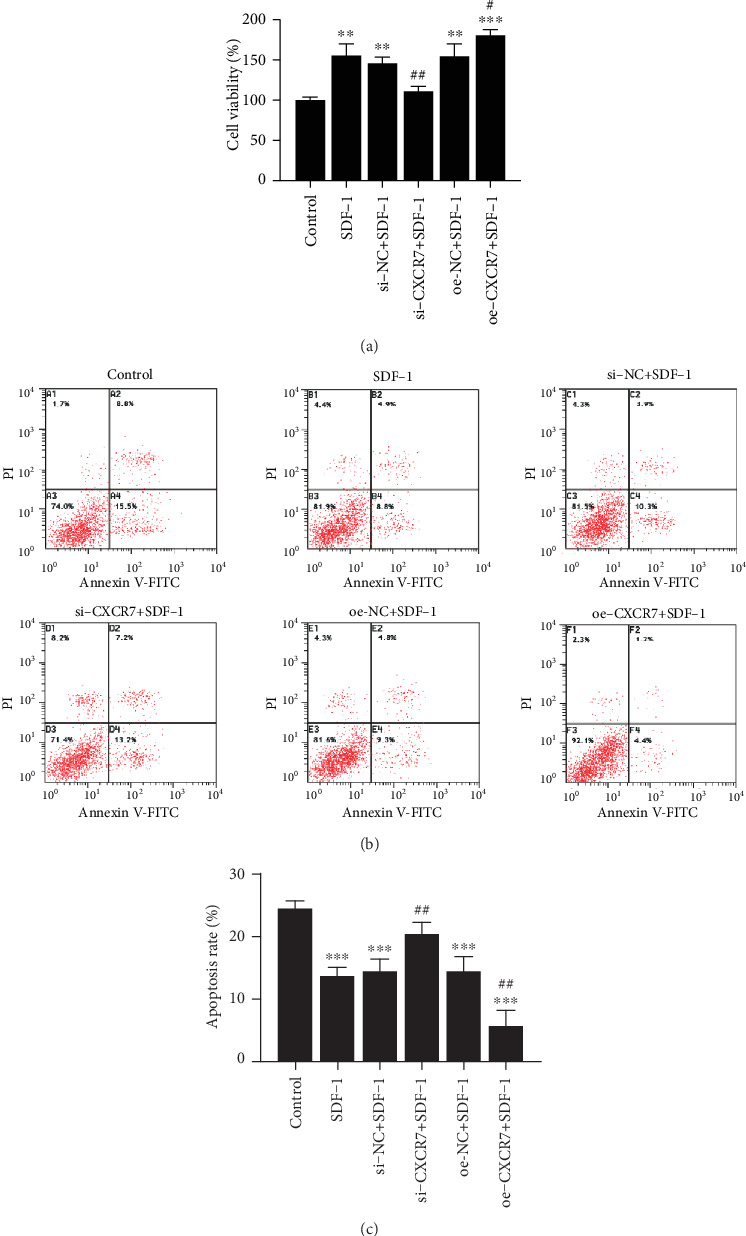
The effects of CXCR7 on the proliferation and apoptosis of HUVECs. (a) Cells proliferation was measured by CCK-8 at 24 h. (b) HUVEC apoptosis was detected by V-FITC and PI staining. (c) The percentage of apoptotic cells was determined and presented as the mean ± SD. si-NC: siRNA negative control group. oe-NC: overexpression negative control group. ^∗∗^*p* < 0.01 versus untreated control group, ^∗∗∗^*p* < 0.001 versus untreated control group, ^#^*p* < 0.05 versus SDF-1 group, ^##^*p* < 0.01 versus SDF-1(100 ng/ml) group.

SDF-1 enhanced cell proliferation of HUVECs by 55.7% (*p* = 0.002) compared to the control cells. We next evaluated the role of CXCR7 in regulating the proliferation of HUVECs. The CXCR7-siRNA cells displayed decreased proliferation ability compared to the SDF-1-treated cells (110.9 ± 5.5 versus 155.7 ± 13.6%, *p* = 0.006), while CXCR7 overexpressed HUVECs showed increased proliferation rates (180.9 ± 6.2 versus 155.7 ± 13.6%, *p* = 0.043). These findings indicate that CXCR7 enhances the proliferation of HUVECs and silencing of CXCR7 inhibits the proliferation ability of HUVECs induced by SDF-1 (Figure 2(a)).

Then, we investigated the potential role of CXCR7 in the survival of HUVECs under SDF-1 treatment by flow cytometry to determine the cell apoptosis. SDF-1 alone prevented the cells from apoptosis (13.6 ± 1.4 versus 24.3 ± 1.3%, *p* = 0.001). Blocking CXCR7 with CXCR7-siRNA promoted the apoptotic effect on HUVECs (20.4 ± 1.8 versus 13.6 ± 1.4%, *p* = 0.006) while upregulated CXCR7 inhibited the HUVECs apoptosis (5.6 ± 2.5 versus 13.6 ± 1.4%, *p* = 0.008). These results suggest that SDF-1 mediates HUVECs survival via CXCR7 (Figures 2(b) and 2(c)).

### 3.3. The Effects of CXCR7 on Migration and Tube Formation of HUVECs

**Figure 3:**
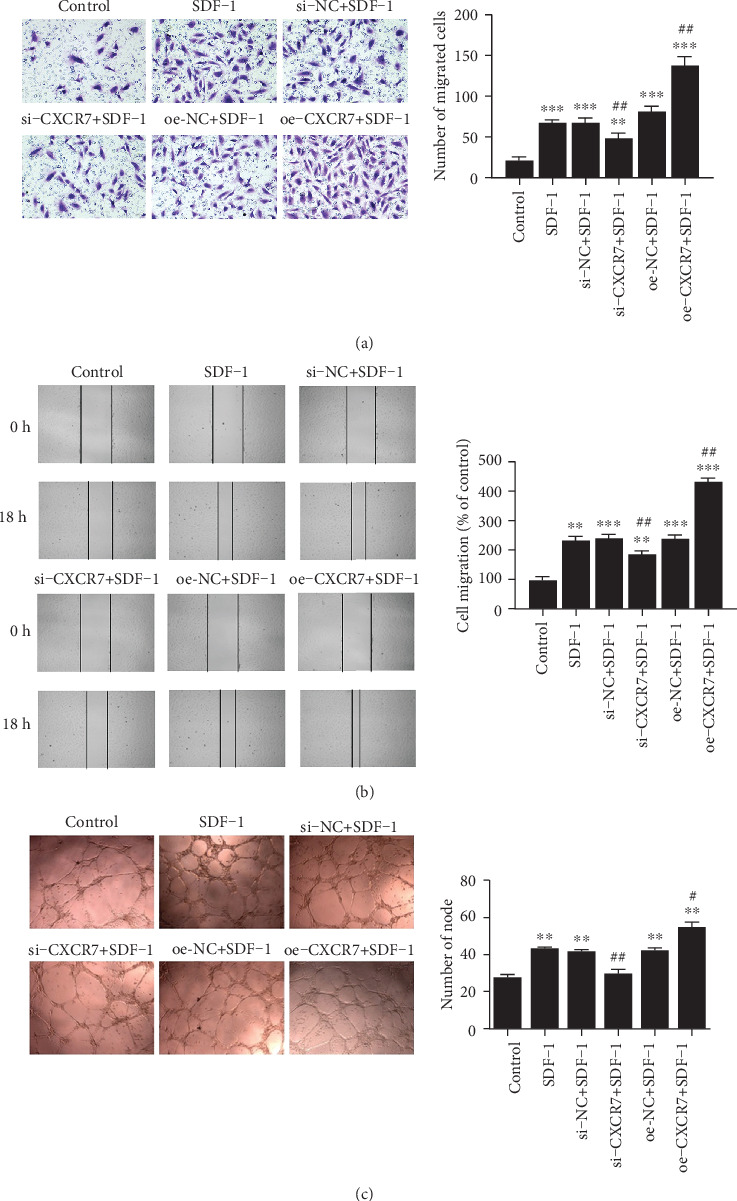
The effects of CXCR7 on migration and tube formation of HUVECs. (a) The migration of HUVECs after different treatments was detected based on the number of migrated cells through the filter inserts. (b) HUVECs after different treatments were scratched and then exposed to SDF-1(100 ng/ml) for 18 h. Wound widths were measured under microscopy and represented as percentage migration considering migration in untreated control as 100%. (c) Cells after different treatments were then exposed to SDF-1(100 ng/ml) for 4 h. Net of tube-like structures were measured for each group. si-NC: siRNA negative control group. oe-NC: overexpression negative control group. ^∗∗^*p* < 0.01 versus untreated control group, ^∗∗∗^*p* < 0.001 versus untreated control group, ^#^*p* < 0.05 versus SDF-1 group, ^##^*p* < 0.01 versus SDF-1 group.

To investigate the contribution of CXCR7 to SDF-1-induced migration of HUVECs, we performed transwell migration assay and scratch wound assay. The migration response to SDF-1 of HUVECs was suppressed by blocking CXCR7 (68.0 ± 3.6 versus 49.3 ± 5.5 cells/filed, *p* = 0.008), while enhanced by overexpressing CXCR7 (68.0 ± 3.6 versus 138.0 ± 10.5 cells/filed, *p* < 0.001) (Figure 3(a)). The same results were obtained by the scratch wound assay (Figure 3(b)). Thus, CXCR7 increases the SDF-1-induced migration of HUVECs.

As shown in Figure 3(c), SDF-1 also boosted tube formation in HUVECs (43.0 ± 1.7 versus 27.3 ± 3.1, *p* = 0.002). CXCR7-siRNA significantly reduced the number of nodes (29.3 ± 4.5 versus 43.0 ± 1.7, *p* = 0.008), while overexpression of CXCR7 enhanced the tube formation ability (54.3 ± 5.8 versus 43.0 ± 1.7, *p* = 0.031). These data suggest that CXCR7 is essential to SDF-1-induced tube formation in HUVECs.

### 3.4. CXCR7 Activates ERK and AKT Pathways but Suppresses the Wnt/*β*-Catenin Pathway in HUVECs

**Figure 4:**
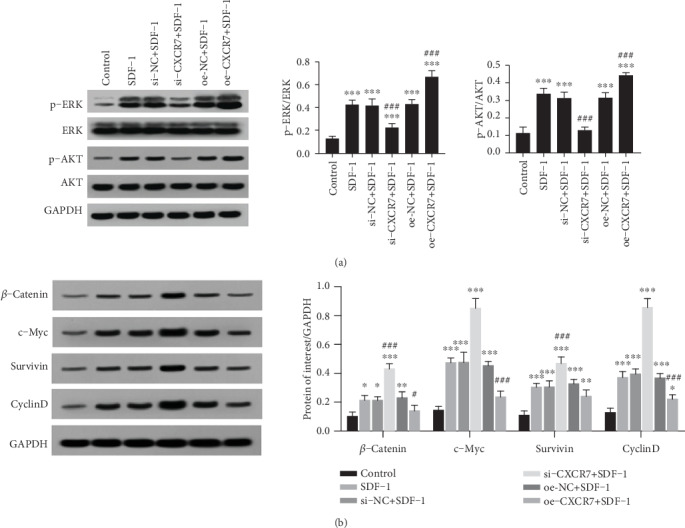
CXCR7 activates ERK and AKT pathways but suppresses the Wnt/*β*-catenin pathway in HUVECs. (a) After cells upregulated or downregulated CXCR7 were treated with SDF-1(100 ng/ml), phosphorylation of ERK1/2 and AKT was detected by western blot analysis. (b) After cells upregulated or downregulated CXCR7 were treated with SDF-1(100 ng/ml), the expression of *β*-catenin and Wnt/*β*-catenin downstream target proteins c-Myc, survivin, and cyclinD1 were detected by western blot analysis. Relative protein levels were normalized to total ERK1/2, AKT, or GAPDH. si-NC: siRNA negative control group. oe-NC: overexpression negative control group. ^∗^*p* < 0.05 versus untreated control group, ^∗∗^*p* < 0.01 versus untreated control group, ^∗∗∗^*p* < 0.001 versus untreated control group, ^#^*p* < 0.05 versus SDF-1 group, ^##^*p* < 0.01 versus SDF-1 group, ^###^*p* < 0.001 versus SDF-1 group.

To investigate the signaling pathway of CXCR7, we assessed total ERK1/2, phospho-ERK1/2, pan AKT, phospho-AKT, and *β*-catenin protein levels in the presence of SDF-1 by western blotting. The phosphorylation of ERK1/2 and AKT increased when CXCR7 was overexpressed, while the level of phosphorylated ERK1/2 and AKT was decreased in CXCR7 downregulated HUVECs (Figure 4(a)). In contrast, the protein level of *β*-catenin decreased when CXCR7 was overexpressed. In addition, the levels of Wnt/*β*-catenin downstream target proteins c-Myc, survivin, and cyclinD1 significantly decreased by upregulating CXCR7 (Figure 4(b)). These data suggest that overexpressed CXCR7 suppresses the Wnt/*β*-catenin pathway in HUVECs.

### 3.5. CXCR7 Inhibits Endothelial-to-Mesenchymal Transition (EndMT) by Wnt/*β*-Catenin Pathway in HUVECs

**Figure 5:**
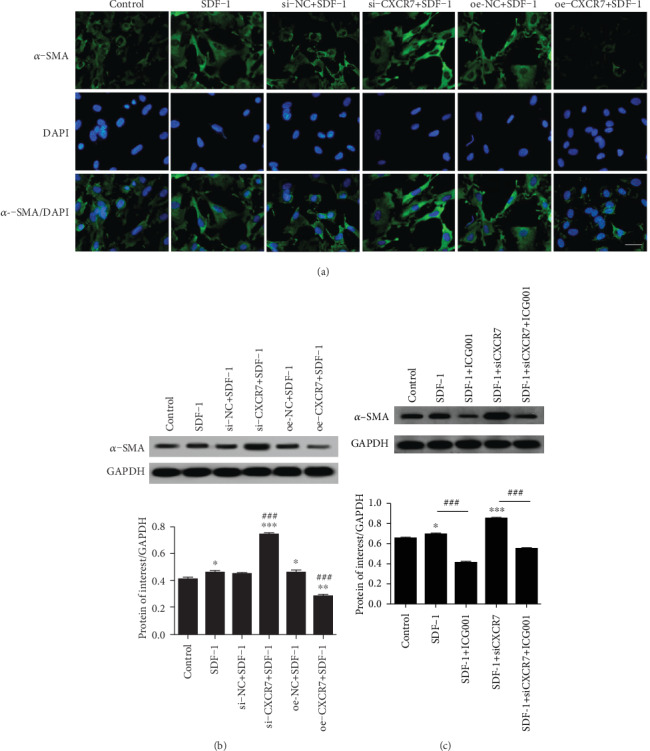
CXCR7 inhibits endothelial-to-mesenchymal transition (EndMT) by the Wnt/*β*-catenin pathway in HUVECs. (a) Cell immunofluorescence staining with *α*-SMA Ab in different treated HUVECs. Scale bar, 50 *μ*m. (b) After cells upregulated or downregulated CXCR7 were treated with SDF-1(100 ng/ml), the protein level of *α*-SMA was detected by western blot analysis. (c) Western blot analysis showed that the expression of *α*-SMA decreased by using the Wnt/*β*-catenin inhibitor (ICG001, 1 *μ*g/ml) in the SDF-1 (100 ng/ml) and CXCR7-siRNA group. si-NC: siRNA negative control group. oe-NC: overexpression negative control group. ^∗^*p* < 0.05 versus untreated control group, ^∗∗^*p* < 0.01 versus untreated control group, ^∗∗∗^*p* < 0.001 versus untreated control group, ^###^*p* < 0.001 versus SDF-1 group.

As CXCR7 ameliorates fibrosis partially due to inhibition *β*-catenin-dependent induction of Jag1 [36], we detected the expression of *α*-SMA by western blotting and immunofluorescence staining in knockdown and overexpressed CXCR7 treated HUVECs (Figure 5(a)). The protein level of *α*-SMA increased in CXCR7 downregulated HUVECs and decreased in CXCR7 upregulated cells (Figure 5(b)). In addition, the expression of *α*-SMA decreased by pretreatment of the Wnt/*β*-catenin inhibitor (ICG001) compared to the CXCR7 downregulated group (Figure 5(c)). These results indicate that CXCR7 inhibits EndMT by Wnt/*β*-catenin pathway in HUVECs.

## 4. Discussion

Angiogenesis refers to many neovascular diseases when the balance is damaged between angiogenic factors and angiogenic inhibitors. In most pathological conditions, angiogenesis is part of a wound healing response culminating, via an angiofibrotic switch, in fibrosis, and scar formation [2]. Our pilot research showed that CXCR7 might be involved in CorNV [23]. However, the role and the mechanism of CXCR7 in CorNV were not fully understood. In this study, we determined the role of SDF-1/CXCR7 axis in angiogenesis in HUVECs.

CXCR7, one of the chemokine receptor for SDF-1, has been demonstrated to regulate the proliferation [11, 37], migration [16, 26, 27, 29, 31, 37], invasion [11, 16, 38], adhesion [11, 21, 37], and tube formation [11, 15, 24, 26, 29, 30, 37, 39] in different cell lines. The function of the CXCR7 depends on the cell type or tissue origin [26]. In this study, we found CXCR7 enhanced the proliferation, migration, and tube formation of HUVECs and inhibited the apoptosis. Consistent with most of the previous researches, these results demonstrate that CXCR7 is essential to the angiogenesis induced by SDF-1. However, we also found that overexpressed CXCR7 reduced the expression of *α*-SMA, suggesting that CXCR7 might attenuate EndMT. Cao et al. [36] reported TC14012, a CXCR7 agonist, promoted lung alveolar repair and reduced fibrosis. Guan and Zhou [40] also proposed that CXCR7 upregulation during angiogenesis was a feedback mechanism to ameliorate pulmonary fibrosis. Together, these results suggest that CXCR7 might inhibit fibrosis during the process of angiogenesis. Besides, TC14012 seemed to have an inhibitory effect on angiogenesis at the late stage of CorNV [23]. Thus, upregulation of CXCR7 might not result in excessive neovascularization along with the inhibition of fibrosis.

CXCR7 belongs to the superfamily of 7-transmembrane G-protein-coupled receptors, but cannot stimulate typical G-protein-dependent pathways [41]. CXCR7 could not only form heterodimers with CXCR4 to recruit *β*-arrestin and activate *β*-arrestin-linked signaling pathways, including the ERK1/2, p38 MAPK, and SAPK/JNK pathways, but also independently activate the AKT and ERK1/2 pathways [42]. In our study, we confirmed CXCR7 simultaneously activated AKT and ERK signaling pathways in HUVECs, which has recently been shown to regulate angiogenesis in tumors [43]. Besides, we unexpectedly found that CXCR7 inhibited Wnt pathway and its downstream target proteins. Wnt signaling is a major regulator of VEGF and plays a role in CorNV [44]. Beyond its role in development, Wnt/*β*-catenin pathway functionally contributes to fibrosis in several organs [45]. The inhibition of the Wnt/*β*-catenin pathway plays a role on antifibrosis [36, 46–48]. A previous study showed that the expression of CXCR7 was controlled by the restriction activation of Wnt/*β*-catenin signaling [49] and conversely CXCR7 inhibited *β*-catenin-dependent induction of Jag1 [36] to ameliorate fibrosis, indicating that CXCR7 and Wnt/*β*-catenin pathways might be functionally linked. In our study, we confirmed that CXCR7 inhibited EndMT via Wnt/*β*-catenin pathway.

Until CXCR7 was discovered, CXCR4 has been considered the exclusive receptor for SDF-1 [8, 9]. Activation of the CXCR4/SDF-1 axis initiates HUVECs migration and angiogenesis via the MAPK/ERK, PI3K/AKT, and Wnt/*β*-catenin pathways [50]. These suggest that both CXCR4 and CXCR7 contribute to the migration and tube formation of HUVECs via ERK and AKT signaling pathways. However, CXCR4 and CXCR7 exert an opposite effect on the Wnt/*β*-catenin pathway in HUVECs. CXCR7 binds to SDF-1 with a higher affinity than CXCR4 and functions as a scavenger of SDF-1 to modulate CXCR4 signaling [51]. Thus, we supposed that continuously overexpressed CXCR7 could inhibit fibrosis along with neovascularization via Wnt/*β*-catenin pathway in vivo.

## 5. Conclusion

In summary, overexpressed CXCR7 might inhibit fibrosis via Wnt/*β*-catenin pathways during the process of angiogenesis in HUVECs. CXCR7 could be a regulator for the pathophysiology of angiogenesis via an angiofibrotic switch.

## Data Availability

The data used to support the findings of this study are available from the corresponding author upon request.
